# Internal and International Migration Across the Urban Hierarchy in Albania

**DOI:** 10.1007/s11113-016-9404-2

**Published:** 2016-07-29

**Authors:** Mathias Lerch

**Affiliations:** Max Planck Institute for Demographic Research, Konrad-Zuse-Str. 1, 18057 Rostock, Germany

**Keywords:** Urbanization, Internal migration, International migration, Urban hierarchy, Urban–rural continuum, Gender, Networks, Albania

## Abstract

The interactions between the processes of urbanization and international migration in less developed and transition countries have important repercussions for socioeconomic development, but are not well understood. Based on the retrospective data from the Albanian Living Standards Measurement Survey 2008, we first assess the geography of migration in terms of the rural–urban continuum, the urban hierarchy and the outside world since 1990. We then investigate the spatio-temporal diffusion of rural-to-urban and international movements using survival models. Results reveal an immediate onset of large-scale rural exodus, despite the post-communist crisis. Internal migrants mainly moved to the capital, bypassing secondary cities, and were predominantly female. Initially, international migrants were primarily men who tended to originate from the main urban agglomerations. The diffusion of opportunities to emigrate down the urban hierarchy and across the sexes then redirected the rural exodus abroad, despite domestic economic development. This evolution in population mobility is related to the gendered patterns and interlinkages of the two flows, as well as to rising inequalities within the urban hierarchy.

## Introduction

Urbanization driven by rural-to-urban migration sustains socioeconomic development in less developed and transition countries through agglomeration economies and improved access to public services (World Bank 2009; World Bank and IMF 2013). Although international migration benefits the sending society through returns of financial, human, and social capital (DeHaas 2010; UNDP 2009), sustained outflows may drain a country’s labor force and development potential. These internal and international movements (hereafter also referred to as out-/in-migration and e-/immigration, respectively) have been studied intensively but independently from each other, and mainly in rural areas. Despite the fact that the majority of the world population is now living in cities, our knowledge about urban emigration remains scant (Fussel and Massey 2004; Hamilton and Villarreal 2011; Randell and VanWey 2015). Theoretical frameworks have not clarified expectations for the interplay between urbanization and emigration. In particular, it is not clear whether we should expect reduced pressure for international migration as rapid development draws rural migrants into domestic cities, or whether international migration is a complement to or substitute for the rural-to-urban flows which have characterized so many developing contexts.

These issues are most specifically addressed in the hypothesis of a mobility transition (Skeldon 1990; Zelinsky 1971). It predicts that rural-to-urban migration accompanies the process of economic development, whereas emigration increases in the take-off phase but levels off at more advanced stages. Yet the role played by social processes and spatial inequalities in development in the course of the mobility transition remains under-appreciated. We test this model in the case of Albania, which has experienced a rapid catch up in urbanization and large-scale emigration since the end of communist rule in 1990 (Carletto et al. 2006; Lerch 2014). We focus on how the spatio-temporal diffusion of rural-to-urban and international migrations has been mediated by the economic and political hierarchy of cities, and by the gendered patterns and interlinkages of the two flows.

The first two sections provide an overview of the Albanian context to set the scene for the country’s post-communist mobility transition. We then present the retrospective survey data and methods, and describe net migration since 1990 across the rural–urban continuum, within the urban hierarchy and to and from the outside world. First internal and international movements were investigated among adult children of household heads in a longitudinal and multivariate perspective. The results reveal how emigration progressively diffused down the urban hierarchy, while the domestic rural exodus started immediately throughout the country on a large scale, despite the economic and political crises in the 1990s. Rural-to-urban migrants mainly moved to the capital, bypassing secondary cities, and were redirected abroad in the subsequent period of economic development. This incongruence with the hypothesis of mobility transition is related to the interlinkages and gendered patterns of the two types of migration, as well as to the inequalities within the urban hierarchy. Finally, some implications for migration theory and policy are discussed.

## The Albanian Context

Albania is a small country (28.784 km^2^) located in the Western Balkans on the margin of Western Europe. The mainly rural society was completely isolated from the outside world under one of the most restrictive communist regimes between 1944 and 1990. The demographic and economic situation was similar to that observed in developing countries. The onset of the fertility transition was late, leading to an average annual population growth of over 2 % during the communist era. After a period of rapid heavy industrialization and major progress in public health and education in the 1950s to 1970s, the economy fell back into crisis in the 1980s. However, the government denied its people the right to emigrate and restrained rural-to-urban migration. In 1989, two-thirds of the 3.2 million inhabitants still lived in rural and mountainous areas (Lerch 2014; Sjöberg 1992). The gross domestic product per capita (GDP) was 936 US dollars,^1^ and basic sanitary infrastructure was lacking in most cities.

Demonstrations and riots against the communist regime in 1990–1991 led to the collapse of the economic and political system. The first multiparty elections were held in conditions of anarchy and in a regional context marked by civil war in the neighboring former Socialist Federal Republics of Yugoslavia. This rupture marked the onset of large-scale population mobility, which was driven by a range of factors. During the period of isolation, foreign countries were idealized, and domestic cities were attributed a higher social status (Caro 2011). The opening up of the society in 1990 removed barriers to aspirations for mobility toward these locations of modernization, especially in the neighboring European Union.

The prohibition of migration for rural inhabitants under communist rule also resulted in increased population pressure on agricultural land (Darques 2004; King 2004). The post-communist process of land privatization then atomized agricultural plots and undermined the predominantly subsistence economy (UNDP 2000). Other push factors were the political crisis and the sharp increase in unemployment after 1990, with the closing down and shrinkage of State industries and administration all over the country (World Bank 2007).

After the ending of their compulsory participation in the labor market enforced under communist rule, and the revival of patriarchal traits of society, women’s economic activity declined particularly severely (INSTAT 2004b). Patriarchal kinship structures, which have remained strong throughout the history of the Western Balkans, filled the vacuum left by the disintegration of state structures in the 1990s, ensuring people’s physical security and providing a social safety net (Fisher 1999; Kaser 2008). These traditional social institutions emphasize a predominant role for men in society and a protective attitude toward women, relegating them to the household sphere.

Albanian migration developed in three phases alongside a discontinuous transition in the social system (Carletto et al. 2006; Caro 2011; King and Vullnerati 2012; Vullnerati 2007). In the first phase (1990–1995) characterized by anarchy during the collapse of the communist regime, there was a development of internal movements from poor and peripheral regions toward the main economic centers. Refugees and male labor migrants moved illegally and temporarily from border areas to the neighboring countries of Italy and Greece (across the Adriatic Sea and over the mountains, respectively). Significant flows of remittances sustained the livelihoods of families left behind, as well as the onset of a rapid economic transition (Agorastakis and Sidiropoulos 2007; INSTAT 2004a; King 2004).

The second period (1996–2001) was marked by a financial crisis related to the collapse of illegal pyramidal banking schemes. (Deposits were attracted at a fast pace by promises of high payments as incentives for further enrollment, until the system became unsustainable.) Large segments of the society lost all their savings, and the situation escalated into renewed social upheavals. Internal migration intensified and international migration diffused into the interior of the country, as well as among women. This was sustained by the attractive force provided by the regularization of Albanians without documents in Italy and Greece (Azzari and Carletto 2009; INSTAT 2014a; Stecklov et al. 2010).

The third period (2002–2011) was characterized by political stabilization, strong economic growth (leading to a GDP per capita of 4087 dollars) and declining levels of rural poverty (from 30 % in 2002 to 15 % in 2008; INSTAT et al. 2009). Surprisingly, emigration continued unabated, despite the financial crisis which has hit the main destination countries since 2008, and the relative resilience of the Albanian economy. Return flows have increased recently but remain comparatively low (INSTAT 2014a).

**Map 1:**
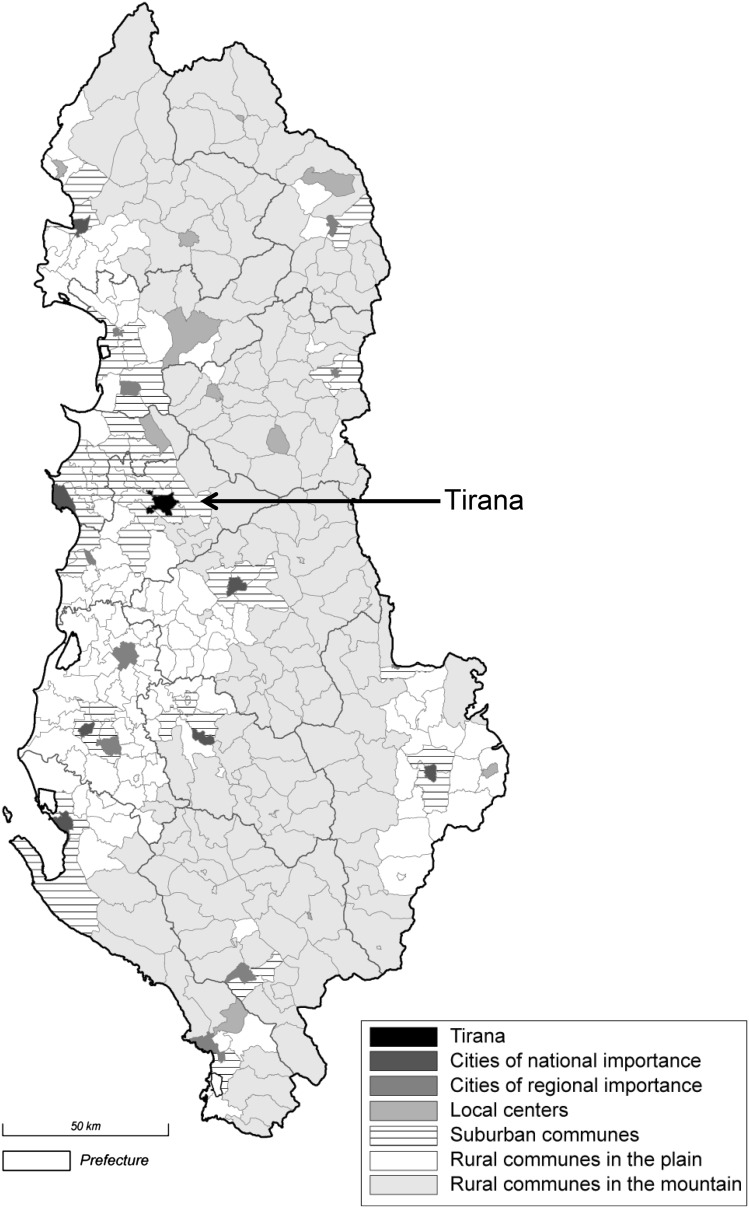
Settlement hierarchy and the urban–rural continuum of Albania, 2001. *Source* Census 2001 and Schuler et al.’s (2010) urban typology

This recent history of population mobility has significantly transformed Albania’s population and economic geography. While the resident population has declined by 10 % since 1989 to 2.8 million in 2011, more than 1.7 million Albanians are living abroad (INSTAT 2012a; Vullnetari 2012a). At the same time, the official level of urbanization has crossed the 50 % bar (up from 34 % in 1990) as a result of a combination of population decline in the countryside and strong urban growth (Lerch 2014). Similar spatial differences in demographic developments across the Western Balkans have been attributed to emigration from remote areas and internal movements toward cities (Bélorgey et al. 2012). Yet it is unclear how these two flows relate to each other, and how mobility patterns differ and are diffused across the urban hierarchy. In contrast to the relatively balanced regional development under central planning in Albania, the countryside and secondary cities have been neglected during the economic restructuring since 1990. The primate-city region constituted by the prefectures of the capital Tirana and of the sea-port-city of Durres (in the West; see Map 1) was the only region with a GDP per capita above the national average in 2011 (INSTAT 2012b). Secondary cities located on the border with the European Union have experienced demographic losses, and the disparities with the capital in terms of living standards, amenities, and infrastructure have widened (Darques 2004; INSTAT 2014b).

Despite the substantial literature on Albanian migration, two key questions remain unanswered. Understanding of the rapid urbanization that started in a period of crisis is hampered by the limited number of studies of internal as opposed to international migration, as well as by their focus on regional rather than urban–rural differences. The unabated trend in emigration during the recent period of development also requires explanation. We address these issues in a transitional perspective of the diffusion of migration across the urban hierarchy over the country’s development trajectory.

## The Mobility Transition and Its Mediating Factors

The theoretical framework builds on Zelinsky’s (1971) hypothesis of a mobility transition. On the basis of a review of historical patterns of population mobility, he conceptualized rural-to-urban and international migration as two spatio-temporal diffusion processes—from central places toward the periphery of a given country—which are related to the modernization of society. He hypothesized that their intensities parallel the sequential increase and decrease in population pressure during the demographic transition, leading to “definite, patterned regularities in the growth of personal mobility through space–time” (Zelinsky 1971, p. 221).

### Diffusion of Migration Across the Urban Hierarchy

Our first set of hypotheses concerns the trends in migration. We expect a diffusion down the urban hierarchy of the opportunities to migrate, which should be more progressive in the case of emigration when compared to out-migration because of the higher barriers to foreign destinations. At advanced stages of development, emigration should decline, while cities continue to absorb internal migrants, until the society becomes predominantly urban. These expectations are consistent with historical and contemporary mobility transitions (DeHaas 2007; Skeldon 1990; Zelinsky 1971). In the early phase of development of a mainly rural society, employment opportunities in rising economic centers attract people from the immediate hinterland. This spatial concentration of financial capacities, international transport connections, and information prompts the onset of emigration from central places, where people are able to overcome the barriers to international mobility. Migratory behavior then diffuses in time and space alongside the regional propagation of development and transport infrastructure down the settlement hierarchy. A differential pace of diffusion of international as opposed to internal migration, and a greater selection from the wealthier social strata in the former than in the latter flow—as found in Mexico and Pakistan (Oda 2007; Poveda 2007)—would confirm the importance of international barriers.

Our second set of hypotheses predicts that the urban hierarchy and the macro-economic context will mediate the spatial pattern of migration. We expect to see a strong focus of out-migration on the capital Tirana because it concentrates export-oriented industries, foreign direct investments, and government subsidies. In developing countries with a strongly differentiated urban hierarchy, we do indeed find the expansion of economic linkages between the primary city and peripheral areas inducing a transformation of short- into long-distance movements, which bypass lower-ranked and less attractive cities (Skeldon 1990). This contrasts with historical patterns of replacement migrations between contiguous regions, in which departures toward more developed areas are compensated for by arrivals from economically less advanced ones—leading to a balanced system of cities (Ravenstein 1885, 1889; Zelinsky 1971).

During the financial crisis in the late 1990s, however, we would expect Albanian out-migrants to bypass all domestic cities and to head toward more attractive foreign destinations, leading to a peak in emigration. Several studies have indeed highlighted the role of the macro-economic context in migrant destination choices, once opportunities to emigrate diffuse and compete with the prospects of internal movement (Massey 1988; Skeldon 2008; Thomas 1973). The subsequent period of economic development should then redirect Albanian migrants toward domestic cities which channel the outflow abroad.

### Diffusion of Migration by Sex

The trends and spatial patterns of migration may also be mediated by social effects. Our third set of hypotheses predicts that gendered patterns of mobility perpetuate migration flows in Albania—especially among women. These expectations are based on historical and contemporary experiences showing that men generally dominate in the early phases of mobility transition, whereas women progressively make up the majority in the domestic and, later on, international flows (Ravenstein 1885; Skeldon 1990). Yet the diffusion of different types of mobility across the sexes has attracted limited attention (Camlin et al. 2014; Reed et al. 2010), and the role of culture remains particularly under-researched (DeWind and Holdaway 2008).

We would expect Albanian men to initiate migration in order to ensure a family’s survival, whereas women’s mobility would be expected to be related to family building and reunification (i.e., a migrant cohort effect). This would be confirmed by a stronger selection of male migrants compared with females among wealthier and better educated subgroups, as pioneer migrants face higher costs, and a higher level of skills raises their probability of finding a job in the upper levels of the urban hierarchy. These hypotheses are informed by the moral primacy of patriarchy in an Albanian context of social upheaval and dangerous illegal border crossing. Similar gendered patterns of migration have also been found in other patriarchal settings (Bohra and Massey 2009; Cerrutti and Massey 2001; Massey et al. 2006). Because of the greater authority of patriarchy in the countryside, we would also expect a weaker selection of migrant women compared to men in rural areas.

### Cross-Migration Network Effects

The inertia of migration streams over time is also influenced by social relations between sending societies and departed members, which enable new candidates to overcome the barriers to mobility (Massey 1990). But our fourth set of hypotheses goes beyond destination-specific migration capital, and predicts that experiences of either internal or international migration among individuals, households, and communities will increase the opportunity to migrate to the alternative type of destination (international and internal, respectively). These expectations are based on the idea of interlinkages between the two flows as reviewed by King and Skeldon (2010), Liang and Chunyu (2013), Mung et al. (1996), and Skeldon (2006).

Individual step-wise movements up the urban hierarchy result in new skills and experience in modern labor markets, as well as access to networks of international transport and information. This may induce subsequent departures to foreign countries, and may help individuals left behind in rural areas to move directly abroad—as has been suggested for Mexico (Davis et al. 2002; Fussel 2004). Moreover, temporary migrants’ accumulation of savings abroad facilitates their resettlement in cities rather than in the peripheral sending areas upon their return, leading to J-shaped residence trajectories. At the same time, the financial capacity to move to cities may increase among those left behind.

Quantitative research in Albania has already documented the gendered patterns of mobility (INSTAT 2004a, 2014a; Stecklov et al. 2010), and migrants’ narratives have highlighted significant interlinkages between internal and international flows (Caro 2011; King and Vullnetari 2003; Vullnetari 2012a). The analyses here investigate how these mediating factors interplay to shape the diffusion of migration across the urban hierarchy.

## Data and Methods

We first defined the urban geography of population mobility since 1990 and then investigated the determinants of internal and international migrations in a longitudinal and multivariate perspective.

### Data, Urban Typology, and Definitions

The main data used for this analysis were taken from the World Bank’s 2008 Living Standards Measurement Survey (LSMS). This provides retrospective information on socioeconomic conditions and on migration within and from Albania for a sample of 3600 households, comprising 14,875 residents, which is representative at the national, regional, and urban–rural levels. Households were randomly selected according to a stratified, two-stage clustered design; the non-response rate was 2 % (more information is provided by INSTAT).

We reconstructed municipality-specific and international residence trajectories for the population aged 15–64 in 1990–2007 (i.e., a synthetic cohort), using the following information. Besides place of residence at survey date, the interviewed household members stated the number of months spent abroad each year since 1990, and the years and places of departure of their last three internal movements. For the household-head’s children living permanently in another place within Albania or abroad (hereafter also referred to as former members), the respondent parent proxied the year and the destination of parental-home leaving, as well as of the subsequent domestic move and of the last three trips abroad. Information on the former members’ internal movements prior to parental-home leaving was not provided. We thus assumed that they did not move independently within Albania until that date, and imputed the internal migration history of the head-of-household for the period of cohabitation. The data may underestimate emigration, as entire families who left Albania before the survey date are not observed.

We linked these residence trajectories to a multidimensional typology of municipalities, which was elaborated based on geo-referenced data from the Albanian Population and Housing Census 2001 (Schuler et al. 2010). This classification defines a political and economic hierarchy of urban agglomerations, which are constituted by an official city-center and surrounding rural (de-facto suburban) communes which meet morphological, demographic, and socioeconomic criteria of urbanization. Three classes of urban centers were distinguished according to administrative importance, population size, and economic centrality: the capital Tirana, located in the center of the country near to the Adriatic coast (on the West), and cities of national and regional importance—hereafter also referred to as secondary and peripheral cities, respectively (see Map 1). Agrarian communes were distinguished according to their accessibility, as proxied by the steepness of the terrain: the plain and the mountains. The remaining remote and small towns (which are inhabited by less than 5 % of the total population) were regrouped in the rural categories. This hierarchical typology of the urban–rural continuum satisfies the need for a functional approach to space in the study of migration, and accounts for urbanization beyond the official city limits. In 2001, in addition to the 42 % of residents living in urban areas, 16 % resided in the cities’ vicinities (Schuler et al. 2010).

International migration was defined as living abroad for at least three months, in order to account for the high seasonality of the phenomenon in the 1990s and to exclude short visits to family members in neighboring countries. Internal migration implies a change in place of residence across the levels of the urban hierarchy, distinguishing the rural mountains and the plain, as well as the suburbs and centers of the three classes of urban agglomerations.

### Analytical Approach

We first described net migration and migration efficiency (i.e., net migration divided by gross-migration) between each pair of the urban hierarchy’s levels and the outside world. We considered all movements of the 1990–2007 synthetic cohort aged 15–64, constituted by the 16,057 current and former household members; place of residence was allowed to vary over time.

The first internal move across the urban hierarchy and the first international departure since 1990 were then analyzed using discrete-time survival models to account for the time-varying nature of the covariates (Allison 1995). In accordance with the transition hypothesis, we investigated the diffusion of both types of migration alongside each other, using separate binary logistic regressions.^2^ The majority of migrants moved only once within Albania and/or abroad.

In order to measure household-level determinants (wealth and migration capital) consistently over time, individuals were characterized by the status of their parental household, for which there is information covering the whole period. We therefore restricted the model’s sample to household heads’ children (i.e., current and former members) and considered only the synthetic cohort aged 15–39, in order to avoid sampling-related underrepresentation of older children in the early 1990s (i.e., children of household-heads aged over 80). This sample selection did not affect the robustness of the (individual-level) results: the conclusions from models including older children and adults interviewed in neo-local households were qualitatively the same (not shown).

Individuals were observed annually starting in 1990 or the year they reached age 15. Exposure ends with the occurrence of the event modeled (first out- and first emigration), truncation at age 40 or in 2007. The sample for the international migration model comprised 4293 men and 3559 women (36,426 and 32,873 person-years), with place of residence within Albania allowed to vary over time (Table 1). The sample for the model of out-migration refers to the same population universe but excludes residents of Tirana city because of the small number of out-migrants; periods spent abroad were also not considered. This gives a sample composed of 3863 men and 3112 women (31,984 and 24,697 person-years, respectively). Suburban dwellers of different city categories were regrouped due to small numbers (tests confirmed a similar spatial pattern of migration).

**Table 1:** Descriptive statistics of the samples for the out- and emigration models, household-heads’ sons and daughters aged 15–39, Albania 1990–2007. *Sources* LSMS 2008, Census 2001

Time-varying characteristics (except *; non-migrants are observed at mid-period, migrants at the time of the event)	Out-migration model		Emigration model	
	Men	Women	Men	Women
Age (average)	23.4	23.6	22.8	23.0
Place of residence				
Tirana	–	–	11.8	14.8
Secondary cities	15.4	19.0	14.3	17.7
Regional cities	17.3	17.5	15.8	15.7
Suburbs	12.3	11.3	11.6	10.0
Rural plain	32.2	29.5	27.9	24.5
Rural mountains	22.9	22.7	18.6	17.3
Total	100.0	100.0	100.0	100.0
Completed education*				
Primary	60.5	54.2	54.7	47.6
Secondary	32.1	33.4	36.6	38.1
Tertiary	7.3	12.4	8.7	14.3
Total	100.0	100.0	100.0	100.0
Number of past emigrations (cumulated and lagged)				
None	94.5	–	–	–
One or more	5.5	–	–	–
Total	100.0	100.0	–	–
Number of past out-migrations (cumulated and lagged)				
None	–	–	91.8	87.7
One or more	–	–	8.2	12.3
Total	–	–	100.0	100.0
Number of past out-migrants from household (cumulated and lagged)				
None	88.8	89.1	85.0	84.0
One or more	11.2	10.9	15.0	16.0
Total	100.0	100.0	100.0	100.0
Number of past emigrants from household (cumulated and lagged, including siblings of the head-of-household and his spouse)				
None	75.9	74.0	68.0	65.1
One or more	24.1	26.0	32.0	34.9
Total	100.0	100.0	100.0	100.0
Average *N* of durables owned by household (cumulative and lagged)	3.8	3.7	4.4	4.6
Average community percentage of out-migrants (1989–2001 cohort rate, net of emigration)	22.6	21.9	19.6	18.3
Average community sex ratio (2001 population by place of residence in 1989)	98.2	97.4	97.6	97.2
Numbers	3863	3112	4293	3559

We specified separate models for men and women, in order to investigate gendered social processes. The statistical significance of gender differences in the effects of each covariate was tested using a 1-degree-of-freedom Wald *χ* ^2^ statistic (Allison 1995). The clustering of siblings at the family level was accounted for by estimating robust standard errors using the generalized estimating equations method (GEE; Liang and Zeger 1986).

The conditional likelihood of a first out- and emigration in a given year is expected to depend on urban hierarchy, sex, socioeconomic status, migration capital, and period. Socioeconomic selection was deduced from the effects of the level of education at survey date and of time-varying household wealth. Wealth was approximated by the cumulated numbers of durables acquired over time, which are normally distributed except in the immediate aftermath of communism (see Table 1 for details and descriptive statistics).

Individual cross-migration capital was measured year on year as the cumulated and lagged number of past movements abroad, for the out-migration model, and across the Albanian urban hierarchy for the emigration model. The numbers of other out- and emigrants from the household were also cumulated year on year. Using data from the 2001 Census, the local prevalence of emigration was proxied by the sex ratio of the municipality-specific populations (which were redistributed according to the reported place of residence in 1989 in order to eliminate the bias related to internal migration). The average of 97 men for 100 women is significantly below the biological norm (i.e., 104–106), indicating high emigration, which in the 1990s predominantly involved men. Relative municipality-specific stocks of out-migrants among these redistributed populations were estimated as well. As the LSMS 2008 did not observe people’s family building process, which is expected to influence women’s mobility in particular, additional descriptive statistics were computed from other sources.

## Patterns of Albanian Migration Across the Urban Hierarchy and Abroad

Following Plane et al. (2005), Fig. 1 presents post-1989 net migration (NM) of the synthetic cohort aged 15–64 between each pair of the urban hierarchy’s levels including the outside world. The width of the arrows represents the relative importance of each net flow in total net migration (only those representing at least one percent are shown). The direction of the arrows indicates the sign of migration balance from the perspective of the lower order settlements. The extent to which this directional focus dominates in the underlying population exchanges is shown by colors that index migration efficiency (MEI). The index ranges between zero, which indicates that the counter flows balance each other, and one, where all migrants move in only one direction.

**Fig. 1:**
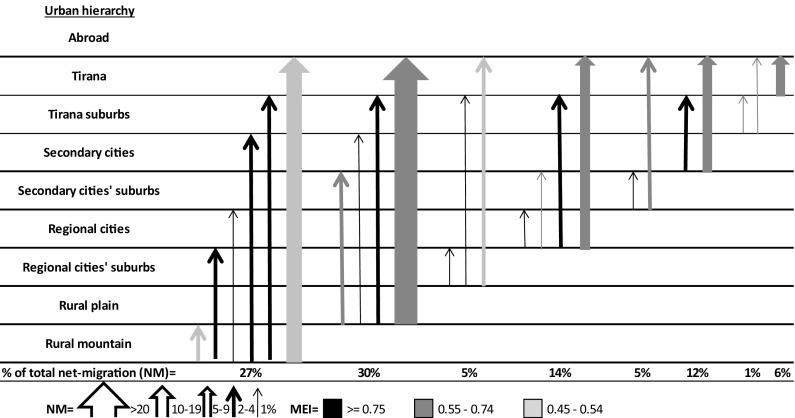
Spatial patterns of net migration across the urban hierarchy and abroad, population aged 15–64, Albania 1990–2007. *Sources* LSMS 2008

All net flows are to the benefit of the upper levels in the urban hierarchy. The majority of the underlying population exchanges are unidirectional, especially the internal flows. This confirms a rural exodus. The geography of departure highlights a role for population pressure and economic deprivation as push factors in lower levels of the urban hierarchy (Schuler et al. 2010). The migration system involves both short-distance movements across local urban borders and long-distance flows linking the lowest to the highest level of the hierarchy, with a strong focus abroad.

Inhabitants of the mountains move to all the upper levels in the urban hierarchy, except secondary city centers. Although the largest population losses are to foreign countries, we show below how this has changed over time. The majority of international migrants originate from the rural plain. Here, domestic movements are more focused on the Tirana agglomeration and secondary cities.

Net migration from the suburbs of regional towns is equally distributed across local urban boundaries and Tirana city, but with a dominant focus abroad. Migrants from regional town centers also short-circuit secondary cities to a certain extent, as they head predominantly to Tirana city and foreign countries. Populations from the suburbs of secondary cities, which are all (but two) located on the borders with the European Union, move into the nearby centers and, particularly, abroad. The secondary city centers’ net flow to foreign countries is also larger than Tirana. The population in the suburbs of the capital essentially moves into the city-center and abroad. Tirana migrants almost exclusively leave the country.

These patterns of population redistribution explain the divergent demographic trends across the urban hierarchy and clarify the processes that underlie the country’s fast urbanization. While internal and international migration depletes the rural population, the only urban agglomeration that experiences a net gain from population mobility is Tirana, where net in-migration is larger than net emigration. Regional and, particularly, secondary cities are affected by demographic losses due to large-scale emigration, and by being bypassed by movers from lower order settlements. Moreover, Tirana appears less attractive than foreign locations, as almost two-thirds of the total rural exodus (from the plain and the mountains) is directed abroad. Cities are also important sources of emigration, accounting for almost half of the total net international flow.

Although emigration clearly out-weighs immigration at all levels of the urban hierarchy, international exchanges were less unidirectional when compared to internal mobility. Many emigrants moved back and forth on several occasions before resettling abroad (Kule et al. 2002). The higher prevalence of this pattern in the peripheral cities’ suburbs and the mountains indicates a more limited financial capacity for relocation and the importance of seasonal agricultural work. The lack of legal channels of mobility also played a role. The numbers of international departures and returnees increased alongside each other during the 1990s. After the regularization programs in the destination countries at the turn of the new century, however, the return flow leveled off, despite a continued rise in emigration (not shown).

## The Gendered Diffusion and Interlinkages of Migrations in Albania

The factors in the spatial diffusion of first out- and emigration were analyzed by step-wise adjustment of the odds ratios according to the urban hierarchy in the multivariate models. We first controlled only for age, and then adjusted for period trends, the effects of socioeconomic characteristics and migration capital, and finally investigated heterogeneous effects of covariates over time.^3^

### Out-Migration

The annual rate of first post-1989 out-migration is significantly higher for household heads’ daughters when compared to sons (2 % against 1 %; Table 2). Age has an inverted U-shaped effect on the likelihood of out-migration (see model M1). The selection of young adults is significantly stronger among women, as compared to men, and accounts for their higher level of out-migration. This is revealed by the inverted sex gradient in the adjusted intercepts of M1 when compared to the crude rate differential.

**Table 2:** Factors determining the first out-migration within the urban hierarchy (discrete-time logistic regression), household-heads’ sons and daughters aged 15–39, Albania 1990–2007. *Source* LSMS 2008

Variables	Men									Women			
	M1			M2			M3			M1		M2	
	OR	S	SD	OR	S	SD	OR	S	SD	OR	S	OR	S
Intercept	−8.07	***	*	−7.78	***	*	−7.86	***	*	−10.38	***	−10.25	***
Age	1.29	***	***	1.30	***	***	1.29	***	***	1.86	***	1.87	***
Squared	0.995	***	***	0.995	***	***	0.995	***	***	0.987	***	0.987	***
Place of residence													
Sec. cities	0.85		***	1.30		***	1.27		***	0.32	***	0.40	***
Reg. cities	1.40		***	0.93		**	0.92		**	0.60	***	0.47	***
Suburbs	1.31			1.38			1.36			1.20		1.23	
Rural plain	1			1			1			1		1	
Rural mountains	2.82	***	***	1.64	**	*	1.66	**	**	1.32	**	1.00	
Completed education													
Primary				0.75	**		0.76	**				0.94	
Secondary				1			1					1	
Tertiary				2.11	***	**	2.10	***	**			1.20	
Household wealth				0.96			0.89	***	***			1.02	
Nbr of out-migrants from household				1.01			0.71	*	**			1.09	**
Nbr of emigrants from household				1.00			0.99					1.01	
Individual emigration capital				1.04			1.35	**					
Community out-migration				1.04	***	**	1.04	***	**			1.02	***
Community emigration (pop SR)				1.01			1.01					1.01	
Period													
1990–2005				0.87			1.26					1.04	
1996–2001				1			1					1	
2002–2007				0.42	***		0.42	***				0.55	***
Period-interacted effects													
Household wealth 1990–2005							1.19	*					
Household wealth 1996–2001							1						
Household wealth 2002–2007							1.14	*					
Individual emigration 1990–2005							0.85						
Individual emigration 1996–2001							1						
Individual emigration 2002–2007							0.43	*					
Nbr of out-migrants from hh 1990–2005							1.76	***					
Nbr of out-migrants from hh 1996–2001							1						
Nbr of out-migrants from hh 2002–2007							1.58	**	**				
Number of events	361			361			361			545		545	
Number of person-years	31,984			31,984			31,984			24,697		24,697	
Number of individuals	3863			3863			3863			3112		3112	
Annual rate of first out-migration (in %)	1.1			1.1			1.1			2.2		2.2	
QICu	3821			3722			3711			5016		4965	

Except education and place of residence, all other individual and family level variables vary over time

*OR* odds ratio, *S* statistical significance, *SD* statistically significant difference between sexes, *SR* sex ratio, *hh* household

* <0.1, ** <0.05, *** <0.01

Out-migration does not significantly differ across the urban hierarchy among men, except for a higher likelihood of leaving the mountains (M1). Women’s likelihood of leaving manifests an unexpected center-periphery gradient, with higher levels in rural areas, particularly in the mountains. Surprisingly, out-migration was high from the beginning of the 1990s and declined after 2001 for both sexes (M2). There is also no spatial diffusion of migration over time, as period-interacted effects of the urban hierarchy are not statistically significant (not shown).

Male out-migrants are positively selected according to educational attainment, whereas no significant differences are observed among women. This is in line with the predominantly economic motivations stated by internal male migrants in the Demographic and Health Survey 2008/2009; by contrast, a majority of women mention family reasons (INSTAT et al. 2010). Our analysis of the Reproductive and Health Survey 2002 (Morris et al. 2005) indeed showed that 89 % of internal migrant women aged between 20 and 29 were married. The two events were interdependent with each other, as 78 % of these women formed their union between 1 year before and two years after they moved. This reveals the importance of marriage migrations.

The effect of wealth is linear and changes direction over time, but is only significant for men (as with education; M3). The period-interacted effects are therefore not shown for women. Male out-migrants were wealthier than non-migrants in the early 1990s and in the 2000s, whereas during the financial crisis in the late 1990s, the likelihood of migration was the highest among the poorest strata.

Domestic family migration capital increased the male and female likelihood of out-migration (M2). Differences by period were only observed for men (M3; period-interacted effects are therefore not shown for women): the effect was inversed in the intermediate crisis period. Thus, a family step-wise migration pattern is confirmed, although the financial crisis may have motivated men with relatives living in cities to stay in the countryside, in an attempt to secure a multi-site livelihood. Moreover, a higher rate of domestic migration from the community also boosts individual out-migration among both sexes, with a significantly stronger effect among men.

Likelihood of out-migration was increased among men having made a higher number of international trips previously, but this is true only for the 1990s. This decreasing incidence of J-shaped residence trajectories over time may be related to a negative selection of returnees, in the context of the opportunities to regularize residence status abroad. The effect of individual emigration experiences could not be tested among women because of the low numbers of returnees.

The control in the model of these spatio-temporal variations in networks totally accounts for the higher likelihood of out-migration observed for women in mountainous areas, and strongly reduces it for men (compare M2/M3 with M1). Thus, chain migrations lifted the barriers to mobility constituted by remoteness, as revealed in migrants’ narratives (Caro 2011).

### Emigration

Unlike in the case of out-migration, the annual rate of first post-1989 emigration is significantly higher for household heads’ sons when compared to daughters (4 % against 2 %; Table 3). Age exerts the same inverted U-shaped effect on the likelihood of emigration among both sexes (see M1).

**Table 3:** Factors determining the first emigration (discrete-time logistic regression), household-heads’ sons and daughters aged 15–39, Albania 1990–2007. *Source* LSMS 2008

Variables	Men									Women					
	M1			M2			M3			M1		M2		M3	
	OR	S	SD	OR	S	SD	OR	S	SD	OR	S	OR	S	OR	S
Intercept	−7.55	***		−6.79	***		−6.78	***		−8.63	***	−7.98	***	−7.95	***
Age	1.48	***		1.53	***		1.53	***		1.54	***	1.64	***	1.64	***
Squared	0.99	***		0.99	***		0.99	***		0.99	***	0.99	***	0.99	***
Place of residence															
Tirana	0.53	***		0.36	***		0.37	***		0.59	***	0.29	***	0.29	***
Sec. cities	0.77	***		0.62	***	*	0.62	***		0.78	**	0.47	***	0.47	***
Reg. cities	0.58	***		0.62	***		0.62	***		0.67	***	0.74	**	0.75	**
Suburbs	1.03		**	0.99		**	0.99		**	0.65	**	0.61	***	0.62	***
Rural plain	1			1			1			1		1		1	
Rural mountains	0.79	***	***	1.00		*	1.00		*	0.48	***	0.72	**	0.73	**
Completed education															
Primary				0.72	***		0.72	***			.	0.61	***	0.61	***
Secondary				1			1					1		1	
Tertiary				0.47	***		0.47	***				0.49	***	0.49	***
Household wealth				1.06	*	*	1.06	*	*			1.19	***	1.18	***
Squared				0.99	**		0.99	**				0.99	**	0.99	**
Nbr of out-migrants from household				0.99		***	0.99		***			1.13	***	1.13	***
Nbr of emigrants from household				1.12	***		1.07					1.08	***	1.10	
Individual out-migration capital				1.59	***	***	1.59	***	***			0.40	***	0.40	***
Community out-migration				0.99	***	***	0.99	***	***			0.96	***	0.96	***
Community emigration (pop SR)				0.996			0.996					0.998		0.998	
Period															
1990–2005				0.58	***		0.66	***				0.59	***	0.69	***
1996–2001				1			1					1		1	
2002–2007				0.89	*		0.89					0.87		0.88	
Period-interacted network effects															
Nbr of emigrants from hh 1990–2005							1.36	**						1.46	**
Nbr of emigrants from hh 1996–2001							1							1	
Nbr of emigrants from hh 2002–2007							1.04							0.97	
Number of events	1586			1586			1586			773		773		773	
Number of person-years	36,426			36,426			36,426			32,873		32,873		32,873	
Number of individuals	4293			4293			4293			3559		3559		3559	
Annual rate of first emigration (in %)	4.4			4.4			4.4			2.4		2.4		2.4	
QICu	12,836			12,617			12,617			7214		6957		6956	

Except education, all other individual and family level variables vary over time, as does community of residence

*OR* odds ratio, *S* statistical significance, *SD* statistically significant difference between sexes, *SR* sex ratio, *hh* household

* <0.1, ** <0.05, *** <0.01

Men’s likelihood is highest in the agricultural plain and suburban areas, followed by secondary city centers and mountainous areas (M1). It is lowest in Tirana and regional cities. Women’s urban gradient is similar, with the exceptions of the significantly lower likelihoods in mountainous and suburban areas. Patriarchal culture has great authority in both of these areas, as out-migrants leaving the mountains have mainly moved to suburban destinations (INSTAT 2014a, b).

**Fig. 2:**
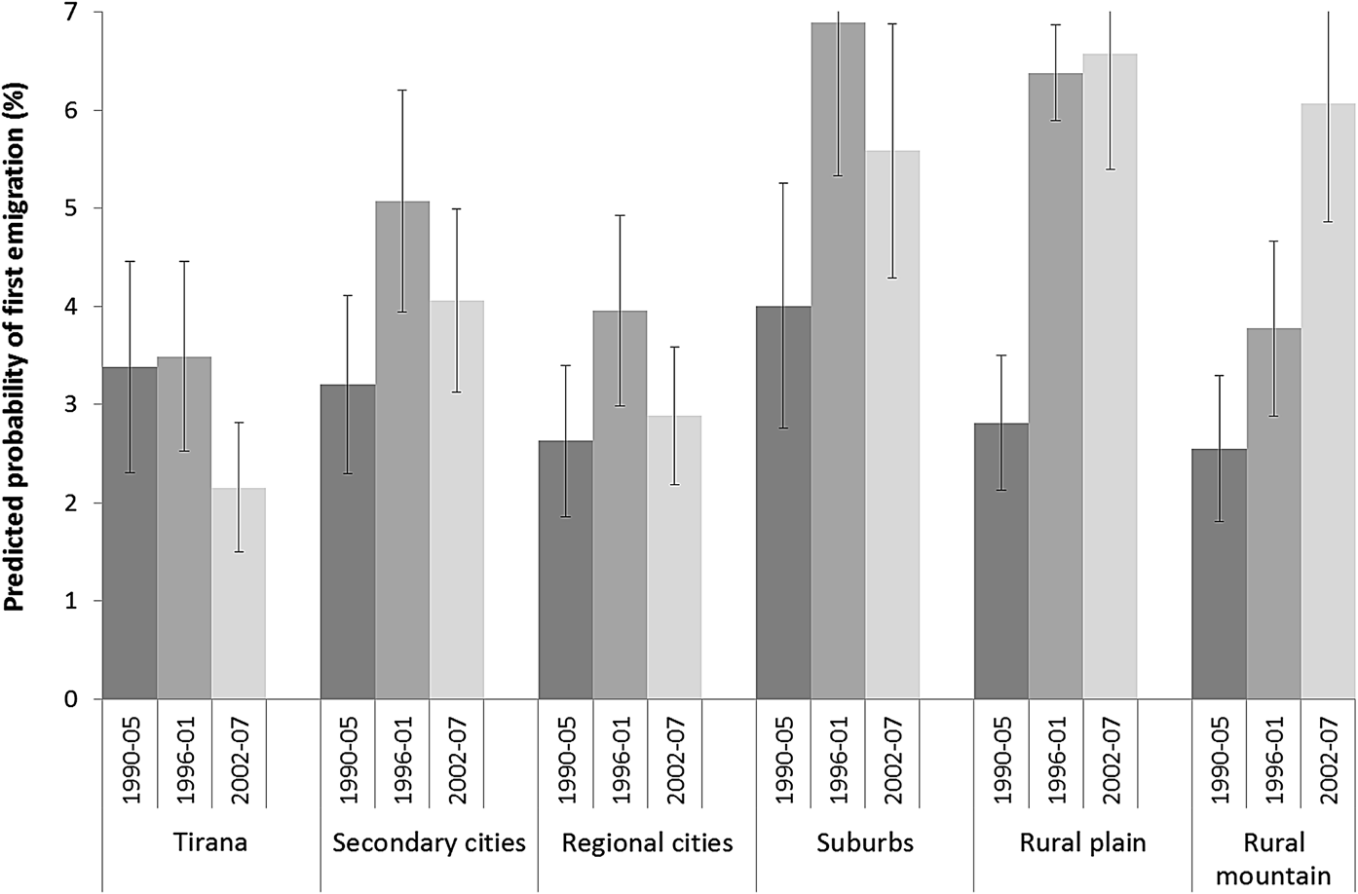
Predicted annual probabilities of first male emigration (from a discrete-time logistic regression), according to place of residence and period, household-heads’ sons aged 15–39, Albania 1990–2007. *Sources* LSMS 2008

The likelihood of emigration increased sharply in the 1990s and did not significantly change in the 2000s among women (M3). Although the likelihood among men has declined recently at the national level, the trend differs across the urban hierarchy. Figure 2 displays predicted annual probabilities (and confidence intervals) of first male emigration according to the urban hierarchy and period, based on a model including only the two variables and their interacted effects.^4^

In 1990–1995, the highest probabilities of male emigration are found in suburbs, Tirana city, and secondary cities. Regional cities and the mountains rank the lowest. Yet this center-periphery gradient is not statistically significant, which may be related to the smaller sample when compared to later periods. Early emigration is also likely to be underestimated in central places, where the subsequent (unobserved) relocation of entire households abroad was more affordable. Emigration from Tirana stabilized during the financial crisis and declined to the lowest levels observed throughout the country in 2002–07, confirming the capital’s increasing economic attractiveness over time. By contrast, the probabilities of leaving other cities increased strongly during the crisis and then declined in regional but not significantly in secondary city centers, where since 1996 the level of urban emigration has remained the highest of all. Suburban areas and the rural plain experienced the sharpest increase in emigration in 1996–2001, which was followed by a plateauing at Albania’s highest levels thereafter. The probabilities of leaving the mountains, by contrast, increased more slowly but continuously through 2002–2007 to similarly high levels. Thus male emigration diffused down the urban hierarchy, with persistently high levels in secondary cities, suburbs, and the countryside.

Conversely, the lower probabilities of female emigration (not shown) rose to a similar extent during the financial crisis throughout Albania, and stabilized or continued to increase in the 2000s. Emigration also remained significantly lower in the mountains when compared with the rural plain and secondary cities.

Male and female international migrants are selected among the median educational and wealth strata of society (M2). While the upper strata may have more domestic employment opportunities, the poorest probably cannot afford to emigrate—as has also been found in Mexico (VanWey 2005). Yet poor men are significantly more likely to move compared to poor women. The limited involvement of highly skilled men in international when compared to internal migration (compare Tables 2 and 3) is surprising, but is consistent with evidence from Mexico and Nepal. There it was attributed to the lower rewards of schooling in the low-paid niches of foreign labor markets (Bohra and Massey 2009; Mora and Taylor 2005; Poveda 2007). This interpretation is supported by qualitative research in Albania (Vullnetari 2012a).

Among women, by contrast, the increased socioeconomic selection of emigrants when compared to out-migrants (compare Tables 2 and 3) is consistent with the higher prevalence of stated economic reasons among those venturing abroad (26 % against 3 %, respectively; INSTAT et al. 2010). Yet work purposes were still mentioned to a lesser extent by emigrant women than by men (94 %). Our analysis of Albanian immigrants in Greece also confirms a role for family motives. According to the public use sample of the 2001 Census (Minnesota Population Center 2014), 79 % of immigrant women aged between 20 and 29 were married, compared to only 35 % of men. Moreover, 44 % of these women married between one year before and two years after they arrived, and more than a quarter had been married for more than one year before arriving (not shown).

A higher number of prior international migrants from the household increases the likelihood of leaving Albania for both sexes (M2 Table 3), which is consistent with a pattern of chain migration observed by Azzari and Carletto (2009) and Stecklov et al. (2010). Family networks abroad were more important for emigration in the early 1990s, when they were scarce (M3). Later on, networks developed rapidly and constituted a defining feature of Albanian society. Emigration capital within the municipality tends to support individual departures, but this was only significant among men when local out-migration was not controlled for in the model (not shown). There seems to be a specialization of migrations at the municipality level, as the likelihood of moving internationally decreases linearly with larger community networks of internal migrants, particularly among women.

The interlinkages between both types of mobility operate at the individual and household level. Having moved across the urban hierarchy increases the likelihood of an international departure among men, thereby confirming step-wise movements (M2 in Table 3). But this effect is inverted among women. They leave Albania to a greater extent directly from internal migrant sending families.

The control in the model of these variations in migration capitals across time and the urban hierarchy intensifies the urban gradient of emigration among both sexes: the likelihood of leaving Tirana or secondary cities decreases, whereas it increases in mountainous areas (compare M2/M3 with M1). Thus the high endowment of international networks explains much of the urban emigration. Specialization in out-migration in the mountainous areas, by contrast, accounts for their lower participation in international movements.

## Discussion and Conclusion

There is increasing recognition that both internal and international movements should be considered as part of better monitoring of trends in developing countries’ population mobility (UNDP 2009). In order to increase our understanding of the interactions between the processes of urbanization and emigration, we tested the hypothesis of a mobility transition in post-communist Albania. Using retrospective survey data, we investigated the geography and spatio-temporal diffusion of migration across the rural–urban continuum, the urban hierarchy, and the outside world.

The results highlight the usefulness of the transitional perspective for understanding the onset and the geographic patterns of migration. Internal mobility clearly was a rural exodus. The onset of emigration in the main urban agglomerations and its diffusion down the urban hierarchy as the process intensified suggest a role for international connections. The selection of migrants among the upper social strata reflected the importance of financial capacities. However, the early trends in Albanian out-migration, its persistent focus toward the capital, and the recent evolution in the internal and international flows challenge the transition hypothesis. The onset of rural exodus throughout the country in 1990 can be explained by the end of totalitarian control of the population. Yet this cannot account for the large scale of rural-to-urban migration despite the crises, nor for its subsequent decline alongside unabated emigration in a favorable economic context in the 2000s. This diffusion of mobility was mediated by three factors revealed by our results, which are backed up by qualitative studies.

First, rural exodus was often a means for international migration, and vice versa. The locational advantages and the early development of international migrant networks in cities attracted men from rural areas to engage in step-wise movements toward foreign countries. Permanent resettlement of families in urban areas also rested on J-shaped residence trajectories which aimed to accumulate savings abroad (Caro 2011; King and Vullnetari 2003; Labrianidis and Kazazi 2006; Vullnetari 2012a). Thus, urbanization in the 1990s was to a certain extent independent of the cities’ economic fortunes.

Second, we found clear gendered patterns of mobility, which are in line with migrants’ narratives about the role of patriarchal culture in structuring the settlement process (King and Vullnerati 2012). This mediated the spatial patterns of migration. Men not only initiated risky (irregular) labor migration to foreign countries, but also pioneered rural-to-urban movements in an uncertain political context. Women mainly moved within the country and appear more responsive to urban-based amenities for family maintenance, as argued also in other countries of the Western Balkans (Bélorgey et al. 2012). Where patriarchal kinship structures exert a stronger authority, emigration was particularly low among women. Yet their out-migration was unexpectedly highest because it was channeled by chain migration and was related to the patrilocal custom of family formation (see also Lerch 2013). Qualitative studies further reveal how male returnees from abroad built or reunified with their families in Tirana, rather than in the remote sending areas (Caro 2011; Labrianidis and Kazazi 2006; Vullnetari 2012a). Therefore, women’s opportunity to out-migrate also depended on the emigration capital of their groom or husband (which could not be observed with our data).

These results lead us to conclude that women’s rural-to-urban migration was higher than international migration in the 1990s because of an existing social safety net in Albanian cities. This protective attitude is also consistent with the fact that women accessed networks and urban-based facilitators of emigration only indirectly, through departed family members. Men, by contrast, accessed them directly through sending communities and individual step-wise movements.

With the sharp increase in and the regularization of men’s emigration during the financial crisis in the late 1990s, family support and protection expanded in neighboring countries. Women’s emigration immediately gained momentum throughout the urban hierarchy, did not level off despite the subsequent economic development, and was again related to family maintenance. Given that marriage has remained a social prerequisite for women planning to leave the country (Caro 2011; Vullnetari 2012b), they may have paradoxically instrumentalized the event to gain more freedom abroad. Socioeconomic selection is stronger for women migrating internationally than internally, which also implies greater bargaining power to escape patriarchal constraints. At the same time, however, it implies higher status on the transnational marriage market. In communities with dominant male emigration, women do in fact marry young and a large share move abroad immediately thereafter (Lerch 2015).

Our results reveal that women are followers in the settlement process, even though they may actively conceive the migration project as a way to ensure a better future for the family (Caro 2011; Vullnetari 2012b), or to contribute to a household’s coping strategy to deal with economic shocks (Stecklov et al. 2010). Thus, women’s decreasing rural-to-urban but rising international migration in the 2000s, alongside the decline in men’s J-shaped residence trajectories, can be interpreted as a shift in the spatial focus of marriage migrations and family reunifications. In other words, emigration in the 2000s has been sustained by a redirection abroad of the migrant cohort effect, which in the 1990s was focused on domestic cities.

Third, the spatial differences in development played a role in shaping Albania’s mobility transition. Given the economic disarticulation of the urban hierarchy, as well as the persistent income gap with Italy and Greece (whose GDP per capita was still seven times higher in the 2000s), people from peripheral areas chose the capital and, increasingly, foreign destinations as the focus of rural exodus. Migrants either bypassed secondary cities on the route abroad, or transited through them and thereby inflated the important share of urban emigration. Future research may investigate the reasons for these persistent step-wise movements, despite the diffusion of international networks throughout the country. Although our data do not cover the period since the 2008 financial crisis, we expect an intensification of the recent patterns observed, not least because of the renewed rise in youth unemployment. The increasing number of mainly male returnees may add to the pool of potential emigrants, as recent flows shift toward new destinations which are less affected by the crisis (INSTAT 2014a, b).

To conclude, the hypothesis of a mobility transition fails to predict the trends in rural-to-urban and international migration in Albania because of its theoretical and empirical basis. The short-term effect of discontinuous trends in development on migrant destination choice cannot be accounted for within the modernization paradigm that underlies the transitional perspective. The crisis-driven substitution of international for internal migration has long-term implications for the course of the mobility transition, through social processes that perpetuate international flows. Subsequent economic growth may therefore not redirect migrants toward domestic cities, especially when the destination and sending countries are spatially close to each other (Skeldon 1997). Prolonged periods of contemporary emigration, despite national development, can also be explained by the higher population pressure and the larger gaps in economic opportunities between sending and destination areas or countries, when compared to historical experiences (DeHaas 2007). The course of mobility transition thus appears to be context- and path-dependent.

This has implications for policies dealing with population distribution and development. As a majority of the Albanian rural exodus was diverted to foreign countries, the recent catch up in urbanization has been driven to a larger extent by rural depopulation, rather than by urban in-migration. Urban primacy was exacerbated by emigration from secondary cities and by rural migrants who bypassed them while heading abroad. Although this constituted a security valve during periods of economic crisis, it is now relocating abroad the potential work force of a rapidly aging population. This has disrupted social life, depleted professional services, and hampered development initiatives as well as cultural conservation in secondary cities. To curb international departures and exploit demographic potential for development within the country, the widening inequalities across the urban hierarchy should be addressed.

## Footnotes

1
http://data.un.org/Data.aspx?d=SNAAMA&f=grID%3A101%3BcurrID%3AUSD%3BpcFlag%3A1.

2 The events were not modeled jointly in a multinomial perspective because the number of plausible alternative destination choices (and locational barriers) to emigration decreases further up the urban hierarchy. Moreover, theory emphasizes the sequencing and interlinkages of the two types of migration (rather than a joint decision-making), while acknowledging their distinctness in terms of locational, legal and cultural barriers (King and Skeldon 2010; Skeldon 2006).

3 We only present the results from those adjusted models that improved in terms of quality (as indicated by a decline in the quasi-likelihood under the independence model criterion, penalized by the number of parameters; QICu; Pan 2001). We tested linear, logarithmic, quadratic, and dichotomous specifications of the effects of continuous variables (i.e., age, wealth, and migration capitals) and retained those providing the best quality of the male models according to the QICu.

4 We consider differences between two probabilities as statistically significant when the estimate of the former does not contain the confidence intervals of the latter (or vice versa).
